# Thermal Performance of Recycled High-Ductility Cementitious Composites Under Various Elevated Temperatures and Cooling Regimes

**DOI:** 10.3390/ma19122533

**Published:** 2026-06-11

**Authors:** Jie Huang, Xinjie Wang, Quanbin Shi, Jiagai Yang, Minqi Hua

**Affiliations:** 1 School of Urban and Rural Construction, Taizhou Polytechnic College, Taizhou 225300, China; 2 School of Urban Construction, Changzhou University, Changzhou 213164, China; 3 School of Civil Engineering & Architecture, Wuhan University of Technology, Wuhan 430070, China

**Keywords:** recycled high ductility cementitious composites, high temperature, cooling regimes, recycled fine aggregate

## Abstract

Driven by the global demand for sustainable construction resources, Recycled High Ductility Cementitious Composites (R-HDCC) exhibit high ductility and cracking resistance, demonstrating significant potential for enhancing structural durability. However, fire resistance remains a critical constraint on its engineering application. To investigate the performance evolution mechanism of R-HDCC after high-temperature exposure, this study examined the effects of different temperatures (200 °C, 400 °C, 600 °C, and 800 °C) and cooling regimes (self-cooling and water-cooling) on R-HDCC. The results indicate that when the temperature exceeded 200 °C, the compressive strength of R-HDCC decreased significantly. At 800 °C, the residual compressive and flexural strengths dropped to below 20% of their initial values. However, water-cooling treatment mitigated the adverse effects on compressive and flexural strength to some extent. In terms of tensile performance, R-HDCC completely lost its functionality at temperatures of 600 °C and above, and the cooling method had minimal influence on tensile behavior. Compared with natural cooling, water-cooling specimens developed fewer microcracks and less interfacial damage, indicating that water-cooling alleviates high-temperature-induced deterioration of the material’s microstructure to a certain degree. These findings provide important insights for the scientific evaluation of the fire resistance of R-HDCC and offer valuable guidance for its practical application.

## 1. Introduction

In recent decades, high ductility cementitious composites (HDCC), a new class of designed cementitious materials that exhibit numerous stable cracking features, have arisen to overcome the intrinsic brittleness and crack susceptibility of ordinary concrete [1,2,3]. Because of its unique fiber reinforcement, optimized microstructure, low brittleness, and self-healing ability, HDCC has the unique capacity to undergo tensile strain hardening [4,5,6,7]. This distinctive behavior facilitated the global use of HDCC, particularly in earthquake design, tunnel construction, strengthening, and repairing engineering [8,9,10]. Nonetheless, micro silica sand was frequently used in place of natural river sand in the cement matrix to improve matrix fracture toughness, encourage the formation of numerous cracks, and promote strain hardening in HDCC [11,12,13,14]. The use of micro silica sand significantly increased the material cost and environmental impact, which limited the wide application of HDCC [15,16].

To expand the applicability of HDCC, researchers have explored a range of alternative materials to micro silica sand [17,18,19,20]. Given the positive environmental effects, recycled fine aggregate (RFA), which is made from crushed and sieved building debris, has become a viable substitute for micro silica sand [21]. This approach has gained rapid development in recent years. Li et al. [22] explored the use of RFA as a replacement for micro silica sand in the preparation of recycled high ductility cementitious composites (R-HDCC). Their study showed that R-HDCC with RFA, still possessed high ductility and multiple unique steady-state cracking properties. Following this, a series of optimization studies on the material composition of R-HDCC have further enhanced its performance, compared with conventional ECC/HDCC, the RFA used in R-HDCC exhibits higher water absorption and contains old, adhered mortar. These characteristics may increase the initial porosity and weaken the aggregate–matrix interface, making the composite more sensitive to thermal dehydration and microcracking. However, the porous nature of RFA may also alter steam transport and moisture redistribution during heating and cooling processes [23,24,25,26,27]. After optimization, the prepared R-HDCC exhibited an ultimate strain of 3.13% and an ultimate stress of 1.88 MPa [24], while its fundamental mechanical properties, including compressive strength (over 40 MPa) and flexural strength (over 4 MPa), remained unaffected.

Nowadays, fire is one of the most common accidents that causes incalculable harm to property and human life [28,29]. Structural component failure caused by fire is becoming an inescapable factor in building engineering design. Due to the widespread usage of HDCC in building rehabilitation and strengthening, fire was an inevitable risk. The addition of polymer fibers, which offered enhanced ductility and other advantageous qualities, was primarily benefits for the R-HDCC [30,31,32,33]. However, under high temperatures, these polymer fibers melted, significantly compromising the performance of R-HDCC and potentially leading to the failure of the entire structure, with catastrophic consequences [30,34,35]. According to the report by Yu et al., the average pore size of R-HDCC before high-temperature deterioration was 0.11 μm. After natural cooling, it increased to 40 μm, while for water-cooling specimens, it reached 35 μm. The larger average pore size distribution in natural cooling specimens resulted in inferior performance. However, Li et al. [30] reported contrary findings: water cooling introduces thermal stress, reducing strength. At 300 °C, the compressive strength of water-cooled specimens was 26–30% lower than that of naturally cooled ones. Crack observations at 600 °C further supported this result. At 900 °C, however, the residual strength difference became negligible, suggesting that thermal damage was offset by new hydration products. The inconsistent findings reported in previous studies may be attributed to differences in matrix composition, fiber type and content, aggregate characteristics, specimen geometry, heating method, holding time, and cooling rate. For dense cementitious materials, rapid water cooling may generate large temperature gradients and induce thermal shock cracking [36]. However, for matrices containing polymer fibers and porous recycled fine aggregates, water ingress and the shortened duration of post-fire high-temperature exposure may limit crack propagation and dehydration damage to some extent. At present, the mechanism of water cooling for R-HDCC incorporating fully recycled aggregates and PVA fibers remains unclear.

This study systematically investigates the high-temperature degradation mechanisms of R-HDCC under different heating temperatures (200 °C, 400 °C, 600 °C, and 800 °C) and cooling regimes (self-cooling (SC) and water cooling (WC)). By comparing the mechanical properties of R-HDCC before and after high-temperature exposure, combined with macroscopic morphological observation and microstructural characterization (including scanning electron microscopy (SEM), thermogravimetric analysis (TGA), and X-ray diffraction (XRD)), the degradation mechanisms of its high-temperature performance are revealed. Specifically, the following three research objectives are proposed: (1) to evaluate the residual compressive, flexural, and tensile properties of R-HDCC after exposure to elevated temperatures; (2) to compare the combined effects of natural cooling and water cooling on residual performance; and (3) to link macroscopic degradation to PVA fiber failure, hydration product decomposition, and microcrack evolution using SEM, XRD, and TGA. This study provides a theoretical basis for the engineering application of R-HDCC.

## 2. Experiment

### 2.1. Materials

**Figure 1:**
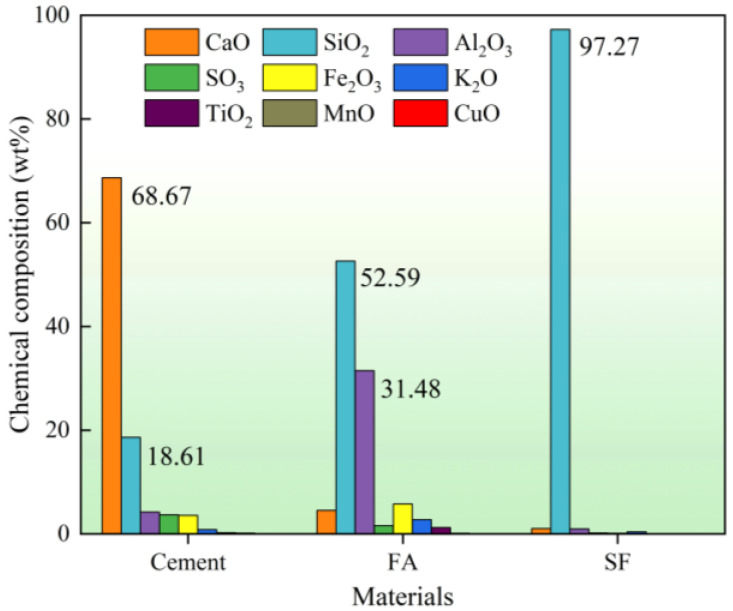
Chemical composition.

**Figure 2:**
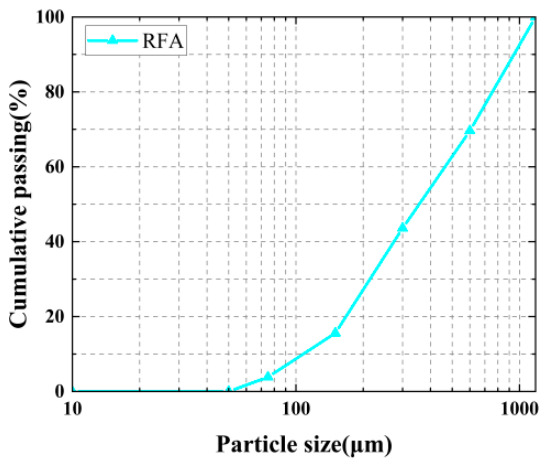
Particle size distribution of RFA.

Class I fly ash (FA), silicate cement (52.5), and silica fume (SF) were utilized as the primary cementitious materials. To further improve the microstructure, minimize crack formation and propagation, and promote secondary hydration, silica fume and fly ash were incorporated into the mix, aiding in the development of additional calcium silicate hydrate (C-S-H) gel from the cement hydration products [37,38]. The chemical composition of these materials is provided in Figure 1 For the aggregate, all fine aggregate in the specimen is recycled fine aggregate, recycled fine aggregate (RFA) was sourced from local construction waste after it was crushed and sieved. The maximum particle size of the RFA was controlled to be less than 1.18 mm, in line with previous studies that aimed for a more optimized particle size distribution [22,24,39]. The particle size distribution of the RFA is shown in Figure 2 with other key properties listed in Table 1. At the same time, 0.3% of high-range water reducer (HRWR) was added to the cementitious material to improve the fluidity and compactness of the specimens.

To ensure that R-HDCC possessed adequate ductility and crack resistance, PVA fibers were incorporated [40]. PVA fibers were selected because their hydrophilic surface promotes mechanical and chemical bonding with the cementitious matrix, which is essential for crack bridging and tensile strain hardening in R-HDCC [41]. However, PVA is sensitive to elevated temperature. During heating, the fibers progressively soften, shrink, melt/dehydrate, and then decompose, causing a rapid reduction in fiber tensile capacity and fiber–matrix bond strength. Consequently, the crack bridging mechanism weakens even before the cementitious matrix completely deteriorates [42]. This characteristic explains why the tensile strain hardening behavior of R-HDCC is more sensitive to temperature than its compressive strength. Table 2 lists the performance specifications of the PVA fibers.

### 2.2. Mixture Design and Specimen Preparation

To ensure adequate ductility and mechanical properties, the mix proportions of R-HDCC were designed based on previous experimental data. Following the findings of [40], the proportions of FA, cement, and SF in the binder were set at 50%, 40%, and 10%, respectively, and the PVA fiber volume fraction was fixed at 2%. Pre-tests were conducted to determine the water-to-binder (w/b) and sand-to-binder (s/b) ratios. Based on a comprehensive evaluation of workability and mechanical properties, the w/b and s/b ratios were finally set at 0.28 and 0.4, respectively. The total binder content was 1227.16 kg/m^3^, yielding an effective mixing water content of 343.60 kg/m^3^. Due to the high-water absorption of RFA (7.69%), an additional 37.75 kg/m^3^ of water was added (based on 490.86 × 7.69%) to compensate for RFA absorption and maintain the designed effective water content. The detailed mix proportions are presented in Table 3.

**Figure 3:**
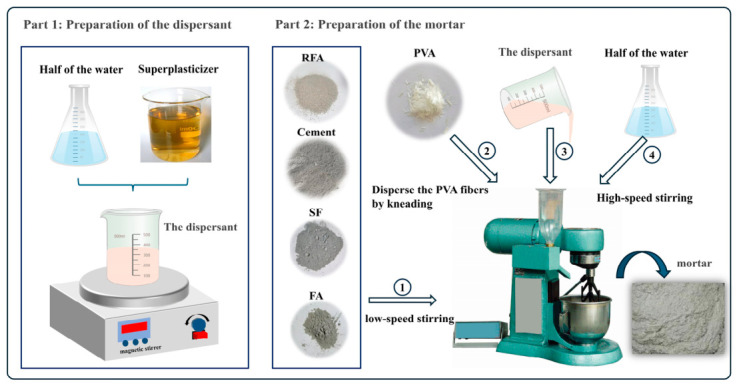
Production of specimens.

In Figure 3, experimental procedure of specimen preparation was carefully planned to ensure the cementitious mixture achieved optimal dispersion. Initially, the mixer was loaded with cement, FA, SF, fibers, and RFA were blended at low speed for 5 min. Besides this, 50% premixed water with HRWR was added to the mixture. Mixing continued for 3 min to ensure adequate mixing. Then, the remaining 50% water was added and stirred for another 3 min to ensure that the fibers were evenly distributed in the mixture. Subsequently, the mixture was poured into the mold, and the mold was placed on a vibration table and shaken for 45 s to effectively eliminate air bubbles and uniformly solidify the sample. To prevent water loss during the curing process, the samples were wrapped in plastic film and cured at room temperature for 24 h. Immediately after demolding, the samples were transferred to a standard conditioning chamber (20 °C and 95% relative humidity). The samples were cured in this environment for 28 days until the tests were performed. All equipment required for this experiment is listed in Table 4.

### 2.3. Experimental Procedures and Methods

According to the standard GB/T 50081–2019 [43] “Standard for test methods of concrete physical and mechanical properties”, cubic specimens of 100 × 100 × 100 mm^3^ were prepared for compressive strength testing. Calculation Formula (1) is as follows:

(1) $$f_{cc}=\frac{F}{A}$$

where

$f_{cc}$—Compressive strength of the concrete cube specimen (MPa), calculated to the nearest 0.1 MPa;

F—Ultimate failure load of the specimen (N);

A—Bearing area of the specimen (mm^2^).

**Figure 4:**
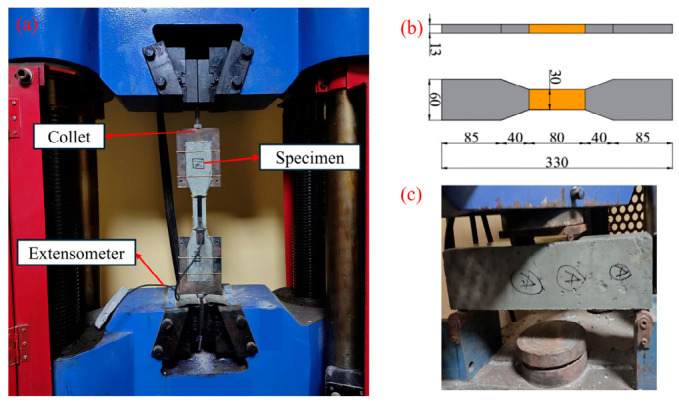
Specific size of uniaxial tensile test specimen (**a**) test instrument (**b**) geometric size (**c**) flexural strength test.

Prismatic specimens measuring 100 × 100 × 400 mm^3^ were prepared for each group for four-point bending tests. The specimen dimensions and testing method for the uniaxial tensile test are shown in Figure 4, and the loading was applied using a universal testing machine at a rate of 0.5 mm/min [44]. Calculation Formula (2) is as follows:

(2) $$f_{f}=\frac{Fl}{bh^{2}}$$

where

$f_{f}$—Flexural strength of concrete (MPa), calculated to the nearest 0.1 MPa;

F—Ultimate failure load of the specimen (N);

l—Span length between supports (mm);

b—Width of the specimen cross-section (mm);

h—Height of the specimen cross-section (mm).

Determination of the compressive/flexural strength value of cube specimens shall comply with the following provisions:

The arithmetic mean of the measured values from three specimens shall be taken as the compressive strength value of that group, calculated to the nearest 0.1 MPa. If the difference between either the maximum or minimum value and the intermediate value exceeds 15% of the intermediate value, both the maximum and minimum values shall be discarded, and the intermediate value shall be taken as the compressive/flexural strength value of that group. If the differences between both the maximum and minimum values and the intermediate value exceed 15% of the intermediate value, the test results of that group shall be considered invalid.

**Figure 5:**
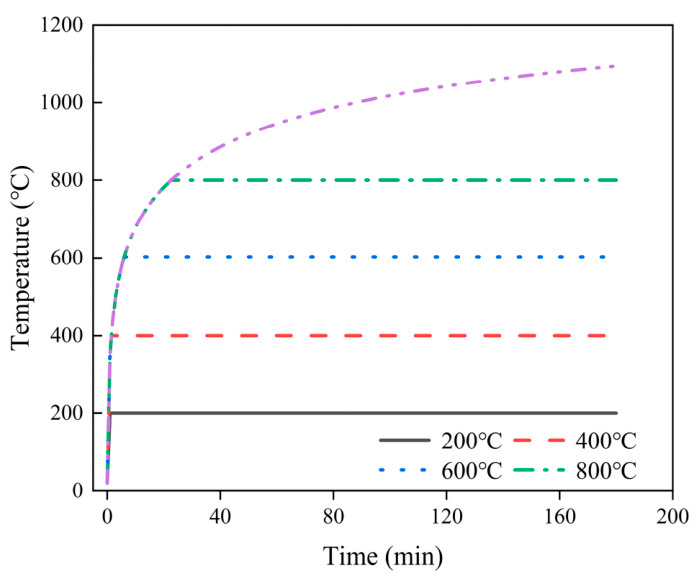
Temperature rise profile of the sample.

This study referred to the standard GB/T 9978.1-2008 [45], “Fire-resistance tests—Elements of building construction—Part 1: General requirements,” and followed the ISO 834 [46] heating curve. In Figure 5, high-temperature tests were conducted at 200 °C, 400 °C, 600 °C, and 800 °C, and the effects of two cooling methods—WC and SC—were compared. First, the specimens were placed in a high-temperature furnace, and each target temperature was maintained for 120 min. After the high-temperature exposure, the specimens were quickly removed using tweezers and subjected to different cooling treatments. For the WC group, the specimens were rapidly transferred with high-temperature tweezers and immersed in a water-filled cooling tank, where they were cooled to room temperature in a short time by the water-cooling system. For the SC group, the specimens were allowed to cool naturally to room temperature beneath the high-temperature furnace. After cooling, subsequent experimental tests (mechanical and microstructural analyses) were conducted on the specimens.

### 2.4. Statistical Analysis

All mechanical tests were performed on three replicate specimens, and results are presented as mean ± standard deviation (SD). Levene’s test was used to assess the homogeneity of variances between groups. For comparisons between the two cooling regimes (SC vs. WC) at the same temperature, independent two-tailed *t*-tests were conducted. A *p*-value < 0.05 was considered statistically significant. The coefficient of variation (CV) was calculated as the percentage ratio of SD to the mean, and the 95% confidence interval (CI) was derived based on the t-distribution. All statistical analyses were performed using IBM SPSS Statistics 27.

## 3. Results and Discussion

### 3.1. Apparent Change

**Figure 6:**
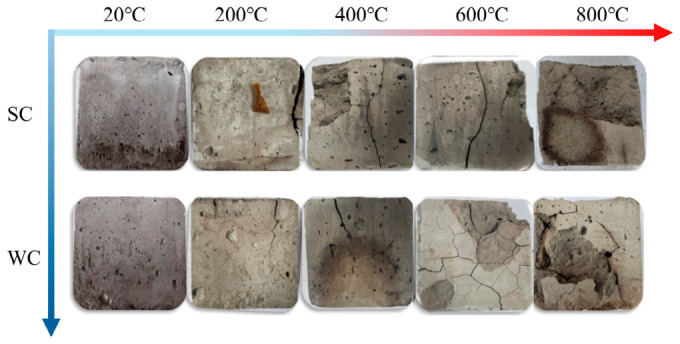
Appearance of R-HDCC under various elevated temperatures and cooling regimes.

The appearance of R-HDCC under different elevated temperatures and cooling regimes is shown in Figure 6. The appearance of R-HDCC demonstrated clear elevated temperature dependency and cooling regime sensitivity. The surface of R-HDCC was relatively smooth and intact, with an overall light gray color and no obvious cracks at 20 °C. When the temperature was set to 200 °C, the R-HDCC exhibited minor discoloration (light yellow) and small cracks under both the WC and self-cooling regimes. The 200 °C target temperature significantly exceeds the chemical decomposition temperature of PVA fibers, resulting in their complete degradation. WC exacerbates thermal shock cracking in the concrete matrix. The presence of yellow colloidal material overflowing on the surface of the self-cooling specimen indicates that the PVA fibers have disintegrated, converting into colloid and escaping from the surfaces. This phenomenon indicated that the PVA fiber has failed at 200 °C, resulting in the high ductility performance loss of R-HDCC at this temperature.

As the temperature increased to 400 °C, a significant increase in surface cracks was observed in the SC group. In contrast, the cracks in the WC group remained relatively minor, suggesting that WC may effectively retard crack formation and possess an anti-cracking effect. In addition, the inner surface of R-HDCC exhibited minute fiber pores, which suggested that the PVA fibers had fully disappeared at 400 °C. This finding corroborated the observations in Section 3.6, where all specimens exhibited characteristics of brittle fracture at 400 °C. At this point, the high ductility performance of R-HDCC was completely lost, and its performance was identical to that of common cementitious materials. When the temperature exceeded 600 °C, the surface of SC R-HDCC fractures enlarged even more, and a sizable region of spalling appeared, creating visible net-like cracks. However, the WC R-HDCC exhibited minor surface damage, characterized by a small patch of localized peeling. At 800 °C, the R-HDCC revealed significant surface spalling and structural damage under both SC and WC regimes.

In conclusion, R-HDCC was significantly impacted by both high temperatures and cooling regimes because of the degradation and failure of the PVA fibers. In contrast to the WC group, which had exceptional fracture resistance and high-temperature stability, the SC group displayed more severe surface cracking and spalling.

### 3.2. Compressive Strength

**Figure 7:**
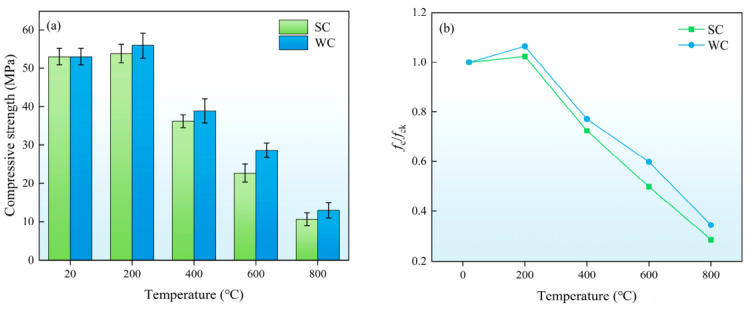
R-HDCC under elevated temperatures and cooling regimes: (**a**) compressive strength, (**b**) relative residual compressive strength.

Figure 7a displays the residual compressive strength of R-HDCC under various elevated temperatures and cooling regimes. The ratio of residual strength to pre-fire strength is known as the relative compressive strength of R-HDCC, and the outcome is displayed in Figure 7b. The compressive strength tended to rise and then fall as the temperature rose, as shown in Figure 7. In particular, when the temperature rose to 200 °C, the compressive strength of the SC and WC specimens increased by 0.8 MPa and 2.9 MPa, respectively, in comparison to those at 20 °C. This phenomenon could be attributed to two reasons. Firstly, the hydration reaction between the cement matrix and the mineral admixtures was further developed at 200 °C, which resulted in more C-S-H and Ca(OH)_2_ hydration products. These products filled the micropores inside the concrete and made the structure denser [47], thereby increasing the compressive strength. Second, the elevated temperature caused the release of bound and free water, inducing a structural change in the R-HDCC matrix toward a denser and more stable crystalline structure. This structural change enhanced internal bonding, improving the resistance of R-HDCC to deformation and damage during compression, and consequently increasing its compressive strength.

However, once the temperature exceeded 200 °C, the compressive strength of the specimens began to decrease sharply. At 800 °C, the relative residual compressive strengths of the SC and WC groups dropped to 20% and 24.3%, respectively. This decline was mainly attributable to the accelerated decomposition of hydration products (especially C-S-H and Ca(OH)_2_) between 200 °C and 800 °C, which led to structural instability of the cement matrix [48]. On one hand, the decomposition of these hydration products reduced chemical bonding, while water evaporation increased, resulting in elevated porosity [49,50,51]. High temperature also induced aggregate phase transformation, altering the crystalline structure of minerals and loosening the microstructure of the cement matrix.

### 3.3. Flexural Strength

**Figure 8:**
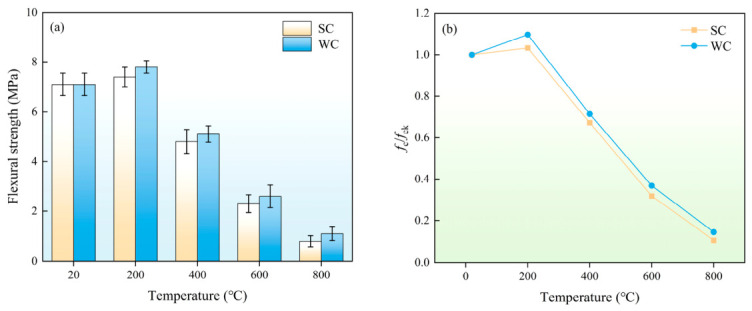
R-HDCC under elevated temperatures and cooling regimes: (**a**) flexural strength, (**b**) relative residual flexural strength.

Figure 8 shows the flexural strength and relative residual flexural strength of specimens under various elevated temperatures and cooling regimes. As the temperature increased, the flexural strength exhibited a trend similar to that of the compressive strength, initially increasing and then decreasing. At 200 °C, consistent with previous findings [52], the elevated temperature accelerated the hydration reaction within the cement matrix. The resulting hydration products filled the voids in the concrete and refined its microstructure, thereby increasing the flexural strength. In addition, the mineral admixtures continued to participate in the hydration reaction at high temperatures, generating more stable hydrates such as C-S-H [49], which enhanced the intrinsic bond strength of the material to further enhance the flexural strength further enhanced.

The breakdown of hydration products and the rise in porosity brought on by high temperatures cause the strength of the sample to decrease. Nonetheless, the findings in Figure 8 demonstrate that at high temperatures, the flexural strength of R-HDCC decreased more than its compressive strength (Figure 7). The SC and WC groups had residual flexural strengths of only 11.3% and 15.4% at 800 °C. Localized damage and fracture extension were more likely to occur on the specimen’s surface tensile zone during the flexural strength test, particularly after high temperatures. The flexural strength deteriorated more severely as a result of the more noticeable localized microcrack extension. Similar to the compressive strength results, after exposure to elevated temperatures, the flexural strength of R-HDCC decreased more for the SC group than for the WC group, especially under low-to-moderate temperature exposures (200–400 °C). WC may allow water to penetrate surface cracks and pores, which could promote limited rehydration of unhydrated particles during subsequent storage [36]. The detailed mechanistic interpretation is presented in Section 3.6.

### 3.4. Statistical Analysis of Mechanical Properties

To evaluate the statistical significance of differences between SC and WC regimes on the residual mechanical properties of R-HDCC, independent two-sample *t*-tests were performed at each temperature level (200 °C, 400 °C, 600 °C, and 800 °C). Each group consisted of three replicate specimens. Levene’s test was used to assess homogeneity of variances prior to the *t*-tests. The statistical results are summarized in Table 5.

As shown in Table 5, no significant differences in compressive strength were found between SC and WC at 200 °C and 800 °C (*p* = 0.378 and 0.198), whereas WC was slightly higher than SC at 400 °C and 600 °C (*p* = 0.048 and 0.033). The mean values at 200 °C were 53.8 MPa (SD = 2.01) for SC and 55.9 MPa (SD = 3.03) for WC; at 400 °C, 36.2 MPa (SD = 1.50) for SC and 38.9 MPa (SD = 1.79) for WC; at 600 °C, 22.7 MPa (SD = 2.42) for SC and 27.6 MPa (SD = 2.49) for WC; and at 800 °C, 10.6 MPa (SD = 1.91) for SC and 12.9 MPa (SD = 1.73) for WC.

For flexural strength, no significant differences were observed between SC and WC at 200 °C, 400 °C, and 600 °C (*p* = 0.237, 0.581, and 0.423), except at 800 °C where WC was significantly higher (*p* = 0.042). At 200 °C, SC recorded 7.4 MPa (SD = 0.31) and WC 7.8 MPa (SD = 0.42); at 400 °C, 4.8 MPa (SD = 0.71) for SC and 5.1 MPa (SD = 0.49) for WC; at 600 °C, 2.3 MPa (SD = 0.30) for SC and 2.6 MPa (SD = 0.50) for WC; and at 800 °C, 0.8 MPa (SD = 0.21) for SC and 1.1 MPa (SD = 0.09) for WC.

These findings demonstrate that the influence of cooling regime on residual mechanical properties is temperature dependent. WC had a certain beneficial effect on compressive strength at 400 °C and 600 °C, and on flexural strength at 800 °C. Overall, WC was beneficial under specific thermal conditions.

### 3.5. Tensile Property

**Figure 9:**
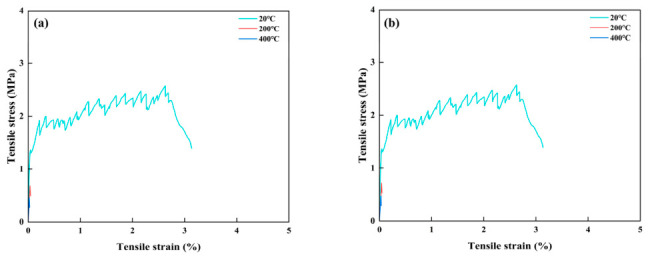
Stress–Strain Curve: (**a**) Self-cooled group, (**b**) Water-cooled group.

Figure 9 shows the stress–strain curves of R-HDCC under various elevated temperatures and cooling regimes. It is evident that R-HDCC lost its strain-hardening properties when subjected to high temperatures. In addition, the cooling method did not have an obvious enhancing effect on the tensile properties of R-HDCC. The PVA fibers deteriorated and thermally decomposed at 200 °C and could no longer provide high ductility to support the tensile properties.

The performance of R-HDCC at elevated temperatures is influenced by multiple factors. Due to the melting and carbonization of PVA fibers at elevated temperatures, their original ability to bridge and restrain cracks is weakened or completely lost, resulting in a significant decrease in ductility and strain-hardening capacity. When the temperature reached 200 °C, the melting and decomposition of PVA fibers meant that the expansion of microcracks within the material could no longer be effectively inhibited; consequently, the specimens at both 200 °C and 400 °C exhibited brittle fracture characteristics. Second, as the temperature further increased, the hydration products in the cementitious composites, particularly the C-S-H gel, began to undergo thermal decomposition. This process gradually loosened the structure of the cementitious composites, significantly reducing their tensile properties. Notably, when the temperature reached 600 °C and 800 °C, the thermal decomposition of C-S-H gel and the evaporation of water further weakened the overall performance of the material [53], ultimately leading to complete specimen failure.

**Figure 10:**
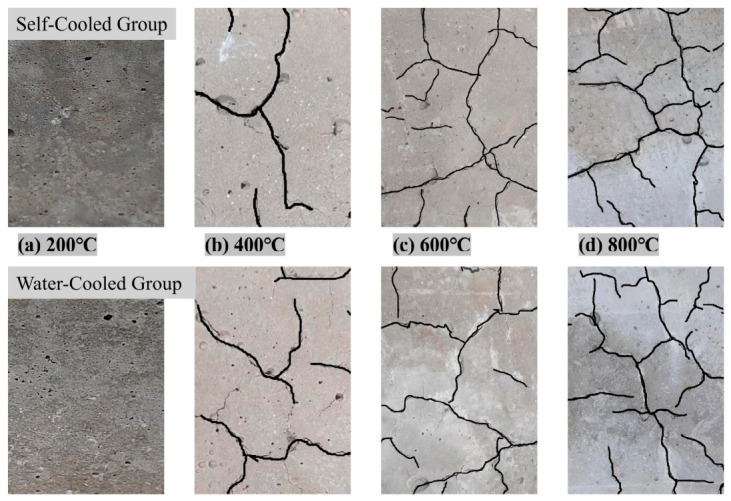
Surface spalling condition of the middle section of dog-bone specimens at different temperatures: (**a**) 200 °C, (**b**) 400 °C, (**c**) 600 °C, (**d**) 800 °C.

As shown in Figure 10, after exposure to these temperatures, the specimens showed severe cracking, surface spalling, and loss of geometric integrity, which made stable gripping and alignment in the uniaxial tensile fixture impossible. After exposure to 200 °C, PVA fibers undergo severe softening and thermal degradation, which reduces their tensile resistance and crack-bridging efficiency. At the same time, dehydration of nearby hydration products and thermal incompatibility between the polymer fibers and cementitious matrix weaken the fiber–matrix interface. As a result, the pull-out resistance and frictional bond along the fiber surface are reduced, and the originally distributed microcracking process changes into localized crack opening. With further temperature increase, the formation of hollow fiber channels and interfacial debonding further accelerates this transition, explaining the brittle response observed at 200 °C and 400 °C and the inability to obtain valid tensile curves at 600 °C and 800 °C [54].

It should be emphasized that the loss of strain hardening at 200 °C has important engineering implications. Residual compressive strength alone may overestimate the post fire functionality of R-HDCC. For applications where HDCC is used for crack control, ductility improvement, energy dissipation, or structural repair, the degradation of PVA fiber bridging at moderate temperature may lead to a loss of the primary functional advantage of the material. Therefore, post fire assessment of R-HDCC should include tensile ductility and crack control capacity in addition to residual compressive and flexural strengths.

### 3.6. SEM Analyses

**Figure 11:**
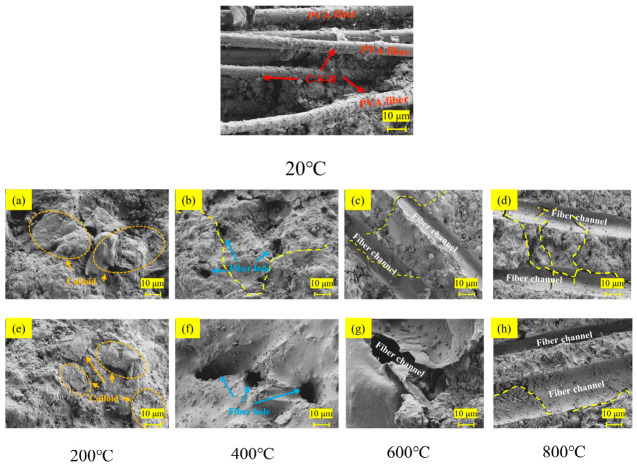
(**a**–**d**) SEM of specimens in the self-cooled group at different temperatures (**e**–**h**) SEM of specimens in the water-cooled group at different temperatures.

As shown in Figure 11, the microscopic SEM of R-HDCC under various elevated temperatures and cooling regimes exhibited a variety of micro-morphologies. As the temperature increased (from 20 °C to 800 °C), the microstructure of the material underwent significant destruction and damage, especially during the cooling process, and the differences in the microdamage exhibited by the samples showed the significant effect of the cooling regime on the microstructure of the material.

When the deterioration temperature was set at 200 °C, the microstructures of the specimens under both WC and SC conditions remained relatively intact, with only a few microcracks. At 400 °C, the SC specimens began to degrade significantly, showing larger cracks and obvious interfacial damage, indicating that the accumulation of thermal stresses caused by slow cooling within this temperature range led to structural damage of the material. In contrast, the WC specimens maintained better structural integrity at the same temperature, with relatively fewer and more widely distributed cracks. At 600 °C, specimens under both cooling regimes showed microdamage. At 800 °C, the structure of the SC specimens was almost completely damaged, with very severe crack propagation and interfacial peeling, indicating that the thermal accumulation effect caused by slow cooling greatly weakened the thermal stability and thermal damage resistance of the material. The WC group specimens also showed significant damage and crack propagation. This indicates that under low-to-medium temperature environments, water spray cooling can mitigate damage to a certain extent. However, under high-temperature conditions, both cooling methods cause severe damage to the specimens [55].

In Figure 11a–d, due to the slow cooling process and insufficient heat release, the SC group exhibited serious microscopic damage. At 400 °C, dense crack networks began to form in the SC specimens, increasing crack depth and leading to more obvious interfacial debonding. In addition, more significant damage was observed around the PVA fiber channels. When the deterioration temperature exceeded 600 °C, the SC specimens showed deeper cracks, greater porosity, and more severe interfacial exfoliation. At the macroscopic level, these microstructural changes manifested as a significant reduction in structural strength, seriously affecting the serviceability of the material. At 800 °C, the structural degradation of the SC group was most severe. Cracks penetrated most of the material, causing substantial damage to its integrity. In Figure 11e–h, although the WC specimens experienced temperature changes, the microstructural damage of the material remained relatively limited under low-to-medium temperature environments [36].

It should be noted that the beneficial effect of WC observed in this study is based on residual mechanical properties and microstructural observations, rather than direct measurements of internal temperature gradients or transient thermal stresses. Therefore, the following explanation should be regarded as a possible interpretation. During WC, the rapid reduction in specimens’ temperature may shorten the period during which the specimens remain at elevated temperatures, thereby limiting further dehydration and thermal decomposition. In addition, water may penetrate into surface cracks and pores, and limited rehydration of unhydrated cementitious particles may occur during subsequent storage. These effects may partially preserve the matrix and interfacial transition zones. However, rapid cooling may also induce thermal shock. Therefore, the net effect of WC depends on the balance between potential thermal shock damage and the reduction in accumulated thermal degradation.

### 3.7. XRD Analyses

**Figure 12:**
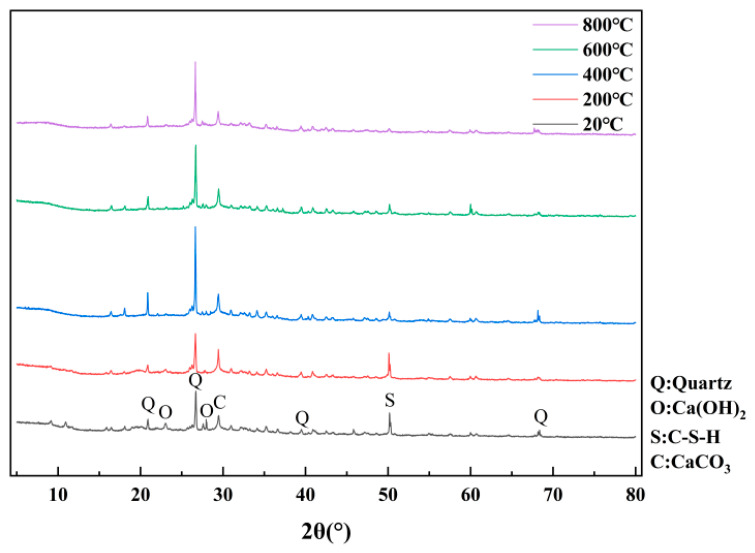
XRD of R-HDCC at different temperatures.

As shown in Figure 12, the XRD analysis of R-HDCC revealed significant changes in its phase composition after exposure to different high temperatures. At 20 °C, characteristic peaks of Quartz, C-S-H gel, Ca(OH)_2_, and CaCO_3_ were all detected. The presence of Ca(OH)_2_ indicated incomplete hydration after 28 days of curing. At 200 °C, the intensity of C-S-H peaks increased, suggesting enhanced hydration of cement and FA, while Ca(OH)_2_ peaks weakened concurrently with strengthened CaCO_3_ peaks. This confirmed that temperatures around 200 °C promote further hydration, leading to a denser matrix and improved mechanical strength.

As temperature rose to 400 °C, the C-S-H peak intensity decreased markedly, indicating decomposition of hydration products, while Ca(OH)_2_ and CaCO_3_ peaks remained stable. At 600 °C, further degradation of C-S-H was observed. By 800 °C, the C-S-H peaks nearly disappeared, and CaCO_3_ peaks were also significantly reduced, reflecting irreversible phase transformation and structural deterioration, consistent with the lowest mechanical strength at this temperature [56].

### 3.8. TGA Analyses

**Figure 13:**
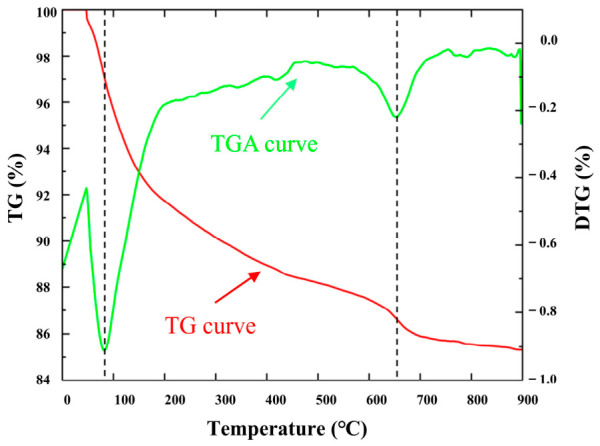
TG and DTG of R-HDCC.

As shown in Figure 13, R-HDCC underwent significant mass loss and phase transitions during the heating process. In the temperature range from room temperature to 200 °C, the first notable mass loss (approximately 8%) occurred, primarily due to the release of physically adsorbed water and some chemically bound water in the C-S-H gel. Meanwhile, a distinct peak appeared around 100 °C in the DTG curve. Notably, the intensity of the XRD peaks increased during this stage, likely because the removal of some free water resulted in a more orderly crystal structure, which led to an increase in the strength of R-HDCC during this period.

In the temperature range of 200–400 °C, the mass loss rate leveled off, with the material losing only about 3% of its mass. This was mainly attributed to the dehydration of Ca(OH)_2_, which corresponded to a reduction in the intensity of the Ca(OH)_2_ characteristic peaks in the XRD pattern. At this stage, the strength of R-HDCC began to decrease due to the breakdown of hydration products and the development of microcracks. As the temperature continued to increase up to 600 °C, the TG curve showed a steady decrease with a total mass loss of about 12%. This was mainly attributed to the further decomposition of the C-S-H gel and the partial decomposition of CaCO_3_, which coincided with the decrease in the intensity of the corresponding characteristic peaks observed in the XRD analysis. Therefore, the strength of R-HDCC continued to decrease significantly, mainly due to the continuous decomposition of the hydration products, which severely damaged the microstructure of the material.

In the temperature range of 600–800 °C, the DTG curve showed a small peak around 700 °C, which may be related to the further decomposition of calcium carbonate. Eventually, the total mass loss reached about 15% at 800 °C. The X-ray diffraction spectroscopy of the material was also very good. At this time, most of the characteristic peaks of the hydration products in the X-ray diffraction spectra were weakened or disappeared, and the intensity of R-HDCC reached its lowest point.

## 4. Conclusions

This study investigated the thermal performance and microstructure of recycled high-ductility cementitious composites under various elevated temperatures and cooling regimes. The findings highlighted the significant impact of temperature exposure and cooling regimes on residual properties of R-HDCC. The conclusions drawn from the experimental results and corresponding analysis are as follows:

(1) The apparent morphology of R-HDCC specimens showed significant temperature dependence and cooling regime sensitivity. Rising temperatures induced surface crack propagation and localized spalling in all specimens. At 200 °C and 400 °C, SC specimens exhibited slightly more severe cracking and spalling than WC specimens, suggesting that water cooling mitigates high-temperature deterioration. At 800 °C, however, no notable difference was observed between the two cooling regimes.
(2) The compressive and flexural strengths of R-HDCC initially increased and then decreased with rising temperature. At 200 °C, enhanced hydration produced a denser microstructure and better mechanical performance. Above 200 °C, decomposition of hydration products and PVA fiber degradation caused a sharp strength reduction. At 800 °C, both residual compressive and flexural strengths fell below 20% of their original values, reflecting severe damage.
(3) After high-temperature exposure, R-HDCC completely lost its tensile strain-hardening capacity. This was primarily due to the thermal decomposition of PVA fibers at 200 °C, which eliminated the fiber bridging effect and caused a sharp deterioration in tensile performance. The cooling regime had little influence on tensile properties, further confirming that PVA fiber degradation was the dominant factor.
(4) Microscopic analysis showed that high-temperature exposure induced microcrack propagation and increased porosity, resulting in mechanical degradation. At 200 °C, WC specimens had lower microcrack density and less interfacial damage than SC specimens, suggesting that water cooling partially mitigates thermal deterioration and facilitates further hydration. At 800 °C, no notable difference in microstructural damage was found between the two cooling methods.

It should be noted that this study primarily evaluates post-fire residual performance through mechanical testing and microstructural observations. Although SEM images reveal fiber channels, microcrack formation, and interfacial deterioration after thermal exposure, direct quantitative measurements of total porosity, crack connectivity, ultrasonic pulse velocity, and permeability were not conducted. Future research should integrate MIP, micro-CT, ultrasonic pulse velocity, and permeability testing to quantify the evolution of pore structure and residual durability of R-HDCC after different thermal and cooling histories. Moreover, considering that rapid cooling may also induce thermal shock, further thermocouple experiments and numerical heat transfer analysis should be carried out to quantitatively obtain internal temperature gradients and transient thermal stress variations, thereby further elucidating the underlying mechanisms.

## Figures and Tables

**Table 1:** Physical properties of RFA.

Apparent Density (kg/m^3^)	Fineness Modulus	Water Absorption (%)
2557	1.71	7.69

**Table 2:** Performance of PVA fibers.

Length (mm)	Elongation (%)	Young’s Modulus (GPa)	Density (kg/m^3^)	Diameter (μm)	Tensile Strength (MPa)
9	7	40	1.29	15.3	1830

**Table 3:** The mixing ratio of experimental specimen (kg/m^3^).

Cement	SF	FA	RFA	Water-o	Water-a	HRWR	PVA
490.86	122.72	613.58	490.86	343.60	37.75	3.68	25.80

Note: water-o is the amount of water used as the baseline, and water-a is the additional water amount.

**Table 4:** Equipment list.

Equipment Name	Model	Manufacturer	City	Country
Cement mortar mixer	JJ-5	Wuxi Jianyi Experiment Instrument Co., Ltd.	Wuxi	China
Ultrahigh-resolution field emission scanning electron microscope	Regulus 8100	Hitachi High-Tech Corporation	Tokyo	Japan
Smart X-ray diffractometer	SmartLab 9kW	Rigaku Corporation	Tokyo	Japan
Microcomputer-controlled electrohydraulic servo universal testing machine	WAW-600C	Jinan Shijin Instrument Co., Ltd.	Jinan	China
Simultaneous thermogravimetric and differential thermal analyzer	LABSYS evo	SETARAM Instrumentation	Caluire	France

**Table 5:** Statistical analysis of mechanical properties.

Temperature	Properties	Cooling	Mean (MPa)	SD (MPa)	CV (%)	95% CI	t-Value	*p*-Value
200 °C	Compressive	SC	53.80	2.01	3.7	[48.8, 58.8]	0.99	0.378
		WC	55.90	3.03	5.4	[48.4, 63.4]		
400 °C		SC	36.20	1.50	4.1	[32.5, 39.9]	1.98	0.048
		WC	38.90	1.79	4.6	[34.4, 43.4]		
600 °C		SC	22.70	2.42	10.6	[18.7, 26.7]	2.51	0.033
		WC	27.60	2.49	9.1	[23.4, 31.8]		
800 °C		SC	10.60	1.91	17.9	[7.0, 14.2]	1.54	0.198
		WC	12.90	1.73	13.2	[9.7, 16.1]		
200 °C	Flexural	SC	7.40	0.31	4.1	[6.7, 8.1]	1.39	0.237
		WC	7.80	0.42	5.1	[6.9, 8.7]		
400 °C		SC	4.80	0.71	14.6	[3.1, 6.5]	0.60	0.581
		WC	5.10	0.49	9.8	[3.9, 6.3]		
600 °C		SC	2.30	0.30	13.0	[1.6, 3.0]	0.89	0.423
		WC	2.60	0.50	19.2	[1.4, 3.8]		
800 °C		SC	0.80	0.21	25.0	[0.3, 1.3]	2.29	0.042
		WC	1.10	0.09	9.1	[0.9, 1.3]		

Note: SC = self-cooling, WC = water-cooling, SD = standard deviation, CV = coefficient of variation, CI = confidence interval, n = 3 per group.

## Data Availability

The original contributions presented in this study are included in the article. Further inquiries can be directed to the corresponding author.
